# Low-dose targeted radionuclide therapy synergizes with CAR T cells and enhances tumor response

**DOI:** 10.3389/fimmu.2024.1355388

**Published:** 2024-03-14

**Authors:** Yanping Yang, Yogindra Vedvyas, Yago Alcaina, Ju Y. Son, Irene M. Min, Moonsoo M. Jin

**Affiliations:** ^1^ Department of Radiology, Houston Methodist Research Institute, Houston, TX, United States; ^2^ Molecular Imaging Innovations Institute, Department of Radiology, Weill Cornell Medicine, New York, NY, United States; ^3^ Department of Surgery, Weill Cornell Medicine, New York, NY, United States

**Keywords:** CAR T cells, targeted radionuclide therapy, ^177^Lu-DOTATATE, SSTR2, solid tumors

## Abstract

Ionizing radiation has garnered considerable attention as a combination partner for immunotherapy due to its potential immunostimulatory effects. In contrast to the more commonly used external beam radiation, we explored the feasibility of combining chimeric antigen receptor (CAR) T cell therapy with targeted radionuclide therapy (TRT), which is achieved by delivering *β*-emitting ^177^Lu-DOTATATE to tumor via tumor-infiltrating CAR T cells that express somatostatin receptor 2 (SSTR2). We hypothesized that the delivery of radiation to tumors could synergize with CAR T therapy, resulting in enhanced antitumor immunity and tumor response. To determine the optimal dosage and timing of ^177^Lu-DOTATATE treatment, we measured CAR T cell infiltration and expansion in tumors longitudinally through positron emission tomography (PET) using a SSTR2-specific positron-emitting radiotracer,^18^F-NOTA-Octreotide. In animals receiving CAR T cells and a low–dose (2.5 Gy) of TRT following the administration of ^177^Lu-DOTATATE, we observed a rapid regression of large subcutaneous tumors, which coincided with a dramatic increase in serum proinflammatory cytokines. Tumor burden was also reduced when a higher radiation dose (6 Gy) was delivered to the tumor. However, this higher dose led to cell death in both the tumor and CAR T cells. Our study suggests that there may exist an optimum range of TRT dosage that can enhance T cell activity and sensitize tumor cells to T cell killing, which may result in more durable tumor control compared to a higher radiation dose.

## 1 Introduction

Chimeric antigen receptor (CAR) T cells are prone to transitioning into an exhausted and dysfunctional state against solid cancers due to various physical and biochemical challenges such as the immune suppressive, hypoxic, and nutrient-deprived tumor milieu, as well as chronic antigenic stimulation from resistant tumors (1, 2). Combination of CAR T cells with oncolytic virus (3, 4), immune checkpoint inhibitors (5), chemotherapy (6), radiation (7, 8), and vaccines (9, 10) have been explored to restimulate dysfunctional T cells or to replace them with vibrant T cells. Among them, radiation holds particular promise due to its established role as a standard of care for more than half of all cancer patients and the ability to precisely control dosage to the target location. However, the mechanism by which radiation augments T cell therapy remains incompletely understood. Nevertheless, it is becoming evident that different radiation doses can produce profoundly distinct biological and physiological outcomes, often exerting opposite effects. For example, cytocidal radiation doses can induce direct tumor cell killing and cause the collapse of tumor vasculature, resulting in cellular damage to both the tumor and tumor-infiltrating lymphocytes (11). On the other hand, low–dose radiation has been shown to activate endothelial cells, promote angiogenesis, reprogram macrophages, and upregulate proinflammatory cytokines and chemokines, recruiting immune cells into the tumor (12–14). Furthermore, low–dose radiation (<5 Gy) has been shown to improve CAR T cell activity by promoting the expression of death receptors in tumors, rendering them more susceptible to soluble factors released by activated T cells (7, 8).

As an alternative to external beam radiation, targeted delivery of radionuclides through endoradiotherapy may also be used to deliver ionizing radiation to augment T cell therapy, particularly for patients with widespread metastatic diseases. However, only a handful of endoradiotherapies have received FDA approval for cancer treatment, starting with ^131^I (15), ^131^I-labeled radioligands (16), and the more recent addition of *β*-emitting radioligands like ^177^Lu-DOTATATE (^177^Lu-DOTA-0-Tyr3-Octreotate) (17) and ^177^Lu-PSMA-617 (18) targeting tumors overexpressing somatostatin receptor 2 (SSTR2) and prostate-specific membrane antigen (PSMA), respectively. This study explores the possibility of harnessing tumor infiltrating CAR T cells to capture radionuclides delivered using FDA-approved endoradiotherapies, aiming to remodel the tumor and stroma to favor immune activity against the tumor. In our previous work, we developed CAR T cells co-expressing SSTR2 to enable positron emission tomography (PET) imaging of CAR T cell distribution in the whole body using ^68^Ga-DOTATATE or ^18^F-NOTA-Octreotide (19, 20). Additionally, we demonstrated the use of SSTR2 as a suicide switch to deliver SSTR2-specific drug conjugates and eliminate unwanted CAR T cells that were causing systemic toxicity (21). In contrast to killing unwanted CAR T cells, this study investigates the effect of low to intermediate doses of radiation delivered by ^177^Lu-DOTATATE to CAR T cells that are largely exhausted and dysfunctional yet proliferating within tumors (22). Due to the *γ*-emission (208 and 113 keV) of ^177^Lu, ^177^Lu-DOTATATE delivery can be imaged by single-photon emission computerized tomography (SPECT) and the absorbed radiation doses at tumor and other major organs can be calculated based on the quantitative SPECT. Our feasibility study indicates that there exists an optimal dose regimen that can stimulate tumor-infiltrating CAR T cells directly, indirectly, or both, thereby regaining anti-tumor immunity.

## 2 Materials and methods

### 2.1 Cell lines

The 293T cell line and the human gastric cancer cell line Hs 746T were purchased from the American Type Culture Collection (ATCC), while the cell line SNU-638 was obtained from the Korean Cell Line Bank (Seoul National University, Seoul, Korea) (23). 293T and Hs 746T were maintained in Dulbecco’s Modified Eagle’s Medium (DMEM, Corning) supplemented with 10% heat-inactivated fetal bovine serum (FBS, GeminiBio). SNU-638 cells were cultured in RPMI-1640 (Corning) supplemented with 10% FBS. To enable bioluminescence imaging of tumor growth in animal models, Hs 746T and SNU-638 tumor cell lines were transduced with a firefly luciferase-F2A-GFP (FLuc-GFP) lentiviral vector (Biosettia, cat. no. GlowCell-16-1). Cells were maintained in a humidified incubator at 37°C with 5% CO_2_ and were routinely tested for mycoplasma using a MycoAlert™ detection kit (Lonza).

### 2.2 Lentiviral vector construction

Lentiviral vectors encoding CARs specific to intercellular adhesion molecule 1 (ICAM-1) and epithelial cellular adhesion molecule (EpCAM) have been previously described (24, 25). The ICAM-1-specific CAR was composed of a c-Myc tag, LFA-1 I domain (F292A), the CD8α hinge and transmembrane domain, 4-1BB co-stimulatory domain and CD3ζ intracellular domain. The EpCAM-specific CAR construct contained from the 5′-LTR end: a c-Myc tag, a single-chain variable fragment (scFv) derived from the anti-EpCAM monoclonal antibody UBS54 (26), the CD8α hinge, the transmembrane and intracellular domains of CD28, and the intracellular domain of CD3ζ. SSTR2 was incorporated at the C-terminus of both CARs using a porcine teschovirus-1 2A (P2A) ribosome-skipping sequence to obtain comparable expression levels of both CAR and SSTR2.

### 2.3 Lentiviral vector production and CAR T cell manufacturing

Lentivirus was produced via transient transfection of 293T cells with transfer plasmid and LV-MAX lentiviral packaging mix (Gibco, cat. no. A43237) using Lipofectamine 3000 transfection reagent (Invitrogen, cat. no. L3000015), following the manufacturer’s instructions. Lentiviral supernatant was collected after 72 hours, filtered through a 0.45 μm filter, and concentrated using PEG-it virus precipitation solution (System Biosciences, cat. no. LV825A-1). The lentivirus was then aliquoted and stored at −80°C.

To manufacture CAR T cells, leukopaks were obtained either from healthy donors (AllCells) commercially or from advanced thyroid cancer (ATC) patients who provided informed consent under the protocol approved by the Weill Cornell Medicine Institutional Review Board (Protocol number, 19-12021154). Primary T cells were isolated through MACS separation using CD4 (Miltenyi Biotec, cat. no. 130-045-101) and CD8 (Miltenyi Biotec, cat. no. 130-045-201) microbeads, and then cultured in TexMACS medium (Miltenyi Biotec, cat. no. 170-076-307) supplemented with 5% human AB serum (Sigma, cat. no. H4522), 12.5 ng/ml of IL-7 (Miltenyi Biotec, cat. no. 170-076-111), and 12.5 ng/ml of IL-15 (Miltenyi Biotec, 170-076-114). After activation with Human T-Expander CD3/CD28 Dynabeads (Gibco, cat. no. 11141D) at a bead:cell ratio of 1:1 (day 0), T cells were transduced twice with lentivirus at a multiplicity of infection (MOI) of 5 on days 1 and 2. Subsequently, T cells were expanded in G-Rex 6M well plate (Wilson Wolf Manufacturing, cat. no. 80660M). On day 10, CAR T cell products were harvested and cryopreserved in a 1:2 mixture of T cell complete growth medium and CryoStor CS10 (STEMCELL Technologies, cat. no. 07930).

### 2.4 Synthesis of radioligands

DOTATATE was purchased from Macrocyclics (Texas, USA) and dissolved in water at 1 mg/ml, and then stored at –20°C in aliquots. ^177^LuCl_3_ was obtained from ITM Pharma Solutions GmbH (Munich, Germany). To prepare ^177^Lu-DOTATATE, ^177^LuCl_3_ was diluted in a solution containing 0.2 M sodium acetate and 0.02 M ascorbic acid to achieve a concentration of 50 mCi/ml. DOTATATE was added into the reaction vial at a ratio of 2 mCi/nmol. The reaction mixture was then incubated in a dry-block heater at 90°C for 30 minutes, followed by cooling to room temperature. High-performance liquid chromatography (HPLC) using a 4.6 mm × 100 mm Symmetry C18 column was employed to determine the radiochemical yield and purity, which consistently exceeded 95%. The final product was diluted with 0.9% saline and passed through a 0.22 μm filter before injection.

NOTA-Octreotide precursor (1,4,7-Triazaclononane-1,4,7-triacetic acid-octreotide) was obtained from ABX advanced biochemical compounds GmbH (Radeberg, Germany) and radiolabeled with ^18^F as previously described (20).

### 2.5 *In vivo* mouse studies

Six to eight-week-old male NOD.Cg-Prkdc^scid^ Il2rg^tm1Wjl^/SzJ (NSG) mice were purchased from The Jackson Laboratory (Stock # 005557). All experimental mice were co-housed in the Animal Core Facility at Weill Cornell Medicine (New York, NY) under specific pathogen-free conditions and provided with sterile food and water. They were maintained at an ambient temperature of 21–27°C and humidity of 40–60%, with a 12 h light/dark cycle. All procedures involving animals were approved by the Institutional Animal Care and Use Committee at Weill Cornell Medicine. Hs 746T and SNU-638 tumor cells were inoculated subcutaneously into the flank of mice in 100 μl of culture media:Matrigel (1:1) solution. Relapsed SNU-638 tumor model was established by subcutaneous implantation of the relapsed SNU-638 tumor fragments into the flank of mice. For the systemic ATC tumor model, ATC001 tumor cells were injected intravenously via tail vein. CAR T cells manufactured with healthy donors were used for the treatment of gastric tumors (Hs 746T and SNU-638), whereas autologous ICAM-1 CAR T cells manufactured from ATC patient T cells were employed in the ATC001 tumor model. All CAR T cells were cryopreserved and adoptively administered via tail vein freshly after thawing. ^177^Lu-DOTATATE was injected intravenously. The treatment time and dose are indicated in the schematics and/or figure legends. Tumor progression was monitored by bioluminescent imaging using the IVIS imaging system (PerkinElmer). Bioluminescence images were acquired 15 min after intraperitoneal injection of 200 μl of 15 mg/ml D-luciferin (Gold Biotechnology) and analyzed using the Living Image v.4.7.2. (PerkinElmer). Whole-body bioluminescence flux was used to estimate tumor burdens. Tumor volume (V) was measured with a caliper on a weekly basis and calculated using the formula V = [length × (width)^2^]/2 for subcutaneous tumor models. Randomization of mice was performed on the basis of bioluminescence imaging or tumor size measurements to ensure equal tumor burden prior to CAR T cell treatment. PET-CT imaging was performed to monitor CAR T cell expansion within the tumor using a micro-PET-CT scanner (Inveon, Siemens) and the Inveon acquisition workplace (Siemens) 2 hours after intravenous injection of ^18^F-NOTA-Octreotide tracer. PET-CT images were analyzed using the AMIDE v.1.0.5. Peripheral blood from subcutaneous Hs 746T model was harvested via retro-orbital blood collection under isoflurane anesthesia at indicated time points. Serum was collected post-centrifugation at 2,000 × g for 15 min at 4°C and stored at −80°C for cytokine analysis. The health condition of mice was monitored on a daily basis by the veterinary staff, independent of the investigators and studies. Mice were euthanized using a CO_2_ chamber, followed by cervical dislocation, when they reached either a maximum tumor size of 3,000 mm^3^ or a humane endpoint, such as losing >25% of body weight or experiencing symptoms of overt disease (e.g., ruffled fur, difficulty with diet, hunched back posture, or abnormal grooming behavior).

### 2.6 SPECT dosimetry

Serial noninvasive SPECT-CT imaging was performed on a SPECT-CT dedicated animal scanner (Inveon, Siemens) to verify tumor targeting and calculate tumor dosimetry following ^177^Lu-DOTATATE treatment. Regions of interest (ROIs) were drawn on the co-registered CT images, and the activities within the spheres were quantified in the reconstructed SPECT images. Non-decay corrected time–activity concentration data were fit to a single exponential model. The cumulated activities were estimated through a combination of mathematical model fitting and area under the curve calculation for the measured data points. Absorbed doses were calculated using the IDAC-dose 2.1 software (27), assuming complete local absorption of the ^177^Lu *β*-emissions.

### 2.7 TIL analysis by flow cytometry

For the ex vivo analysis of tumor-infiltrating lymphocytes (TILs), tumor tissues from the lungs of the ATC001 tumor model were collected 29 days following T cell treatment. Tumors were dissected into small fragments and digested in RPMI-1640 medium containing collagenase type IV (200 U/mL, Gibco) for 1 hour at 37°C. The samples were then dissociated using a serological pipette and filtered through a 70 μm cell strainer to generate single cell suspensions. TILs were isolated through MACS separation using human CD4 (Miltenyi Biotec, cat. no. 130-045-101) and human CD8 (Miltenyi Biotec, cat. no. 130-045-201) microbeads. Subsequently, the samples were stained with Pacific Blue-conjugated human CD3 antibody (Biolegend, clone HIT3a), FITC-conjugated c-Myc antibody (Miltenyi Biotec, clone SH1-26E7.1.3), and APC-conjugated human SSTR2 antibody (R&D Systems, clone 402038). A fraction of the TIL sample was cultured in complete T cell growth media for 24 hours and then analyzed by flow cytometry with the same settings as above. The pre-infusion CAR T cell product was also assessed and served as the baseline for the expression levels of CAR and SSTR2 on the cell surface. Cell staining was performed at room temperature in the dark for 15 minutes, followed by two washes and a staining with propidium iodide before analysis on a Gallios flow cytometer (Beckman Coulter). Flow cytometry data were analyzed using FlowJo v.10.8.1 (Tree Star, Inc.), with CD3^+^ cells gated as T cells.

### 2.8 Cytokine quantification

Cytokine levels in clarified mouse plasma were quantified using a custom LEGENDplex™ panel (BioLegend), following the manufacturers’ instructions. Plasma from untreated mice was used as the control. Cytokine concentrations were calculated using a standard curve (standards provided within the kit).

### 2.9 Statistical analysis

Statistical analysis was performed using Prism 10 (GraphPad, Inc.). An unpaired, two-tailed student’s t-test was performed to evaluate differences between groups. Mouse survival curves were generated using the method of Kaplan–Meier, and the significance was analyzed with the log-rank (Mantel–Cox) test. *P* < 0.05 was considered statistically significant.

## 3 Results

### 3.1 PET-CT imaging of CAR T cells to guide the TRT treatment

**Figure 1:**
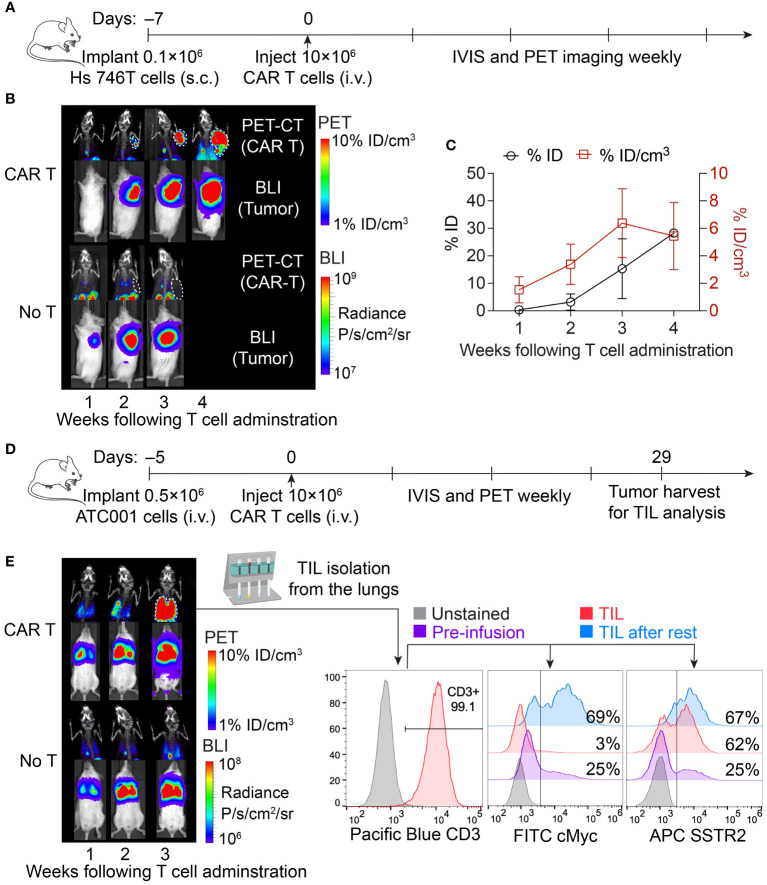
Longitudinal imaging of CAR T cells in subcutaneous and systemic xenograft tumor models. **(A)** Schematic of the subcutaneous Hs 746T model. NSG mice were subcutaneously implanted with 0.1 × 10^6^ Hs 746T cells and treated on day 7 post-xenograft with ICAM-1 CAR T cells (10 × 10^6^ cells/mouse) via tail vein. **(B)** Representative PET-CT imaging of CAR T cells and concurrent bioluminescence imaging of tumor cells over 4 weeks post CAR T cell treatment. **(C)** Quantification of tumor uptake of ^18^F-NOTA-Octreotide in CAR T-treated mice (*n* = 3 biologically independent mice). **(D)** Schematic of systemic ATC001 model. NSG mice were inoculated with 0.5 × 10^6^ ATC001 tumor cells intravenously and treated on day 5 post-xenograft with ICAM-1 CAR T cells (10 × 10^6^ cells/mouse) via tail vein. **(E)** PET-CT imaging showed the expansion of CAR T cells in the lungs where tumor burden was detected by concurrent bioluminescence imaging. The expression of CAR (based on cMyc tag staining) and SSTR2 in TILs isolated from the lungs was analyzed by flow cytometry.

To leverage the capabilities of CAR T cells in delivering targeted radiation via SSTR2-specific ^177^Lu-DOTATATE, it is essential to monitor the expansion of CAR T cells within the tumor. This tracking is crucial for identifying the optimal timing of ^177^Lu-DOTATATE treatment. To this end, we performed longitudinal PET-CT imaging of ICAM-1 CAR T cells using ^18^F-NOTA-Octreotide in a Hs 746T gastric cancer model, where tumor cells lack overexpression of SSTR2 on their surfaces (**Figures 1A–C**). Maximum intensity projection PET images revealed the specific uptake and retention of ^18^F-NOTA-Octreotide in tumors treated with ICAM-1 CAR T cells. The total uptake of ^18^F-NOTA-Octreotide (% ID) displayed a rapid and continued increase as tumors progressed. Meanwhile, the standardized uptake (% ID/cm^3^) increased rapidly in the first 3 weeks, maintaining a range between 3–7% ID/cm^3^ thereafter. In contrast, no detectable signals were observed in Hs 746T tumors from control cohort of No T mice that did not receive CAR T cell treatment (**Figure 1B**). These results suggest that ^177^Lu-DOTATATE treatment could be guided by ^18^F-NOTA-Octreotide PET imaging, allowing treatment initiation at a point with substantial CAR T cell infiltration within tumors (>3% ID/cm^3^).

We further validated the specific uptake of ^18^F-NOTA-Octreotide by autologous tumor-infiltrating CAR T cells in a systemic tumor model of advanced thyroid cancer, where tumor burden was observed in the lungs following intravenous injection of tumor cells (**Figures 1D, E**). Additionally, we isolated TILs from the lungs and observed a loss of surface CAR expression within TILs, as indicated by reduction of cMyc tag staining (**Figure 1E**). The downregulation of CAR expression following tumor antigen stimulation has been recognized as one of the major limiting factors of CAR T cell functionality and antitumor efficacy within the tumor microenvironment (28, 29). Consistent with the findings from others, we observed recovery of CAR expression in TILs following a 24-hour resting period *in vitro* without tumor stimulation (30). In contrast, we did not detect any significant downregulation of SSTR2 expression, indicating that the loss of surface CAR was primarily driven by antigen stimulation (**Figure 1E**). This sustained expression of SSTR2 underscores its value in tracking CAR T cells and the image-guided delivery of ^177^Lu-DOTATATE for TRT.

### 3.2 Specific delivery of ^177^Lu-DOTATATE to tumor-infiltrating CAR T cells and SPECT-based dosimetry

**Figure 2:**
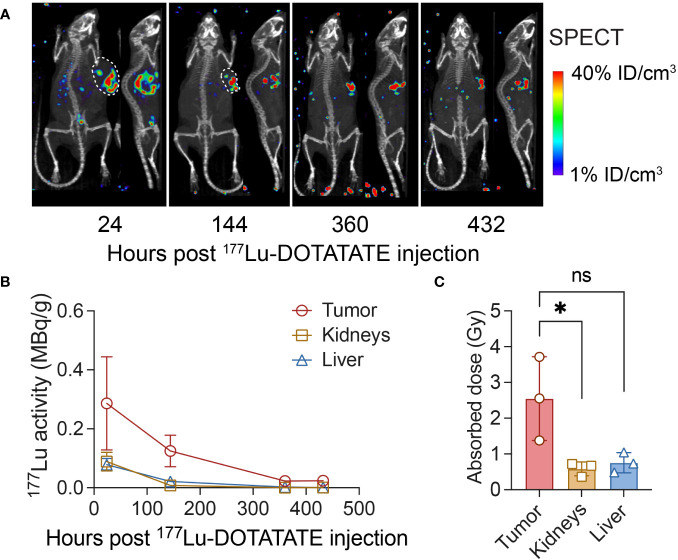
SPECT-CT imaging following ^177^Lu-DOTATATE treatment and dosimetry. **(A)** NSG mice were subcutaneously implanted with 0.1 × 10^6^ Hs 746T cells and treated on day 7 post-xenograft with ICAM-1 CAR T cells (10 × 10^6^ cells/mouse) via tail vein. On day 28 post CAR T cell treatment, mice received a single dose of ^177^Lu- DOTATATE (7.4 MBq) intravenously and were serially imaged using SPECT-CT at indicated time points. **(B)** The effective non–decay corrected ^177^Lu activities derived from SPECT image analysis (as MBq/g). **(C)** Absorbed doses estimated in tumor, kidneys and liver. *n* = 3 biologically independent mice. Statistical significance was determined using the unpaired, two-tailed student’s t-test. *P < 0.05; ns, not significant.

In the study using Hs 746T gastric cancer model (as illustrated in **Figure 1A**), we administrated 7.4 MBq of ^177^Lu-DOTATATE to mice on day 28 following CAR T cell treatment. Subsequently, we conducted serial SPECT-CT imaging at 24, 144, 360 and 432 hours post intravenous injection of ^177^Lu-DOTATATE. The SPECT-CT images demonstrated a highly selective localization of ^177^Lu-DOTATATE within the tumor tissue, with minimum background levels recorded in the rest of the body (**Figure 2A**). At 24 hours post-injection, the effective ^177^Lu activity in the tumor was measured to be 0.29 ± 0.16 MBq/g, corresponding to an uptake of 4.29 ± 2.37% of injected dose of ^177^Lu-DOTATATE per gram of tumor (**Figure 2B**). Furthermore, the non-decay corrected activity in tumor was 0.12 ± 0.05 MBq/g at 144 hours post-injection, representing a 71% retention of ^177^Lu activity after accounting for the physical decay of ^177^Lu (0.17 MBq/g). This sustained retention of ^177^Lu activity in the tumor suggests minimal biological or metabolic clearance of ^177^Lu from the tumor and alludes to survival of CAR T cells. The cumulated absorbed doses for the tumor, liver, and kidneys were calculated to be 2.55 ± 1.17 Gy, 0.58 ± 0.19 Gy, and 0.75 ± 0.28 Gy, respectively (**Figure 2C**). Importantly, the absorbed dose in the kidneys (0.75 Gy) was well below the threshold nephrotoxicity dose (24 Gy) in mice (31).

### 3.3 Low-dose TRT enhances CAR T cell activity and improves anti-tumor response

**Figure 3:**
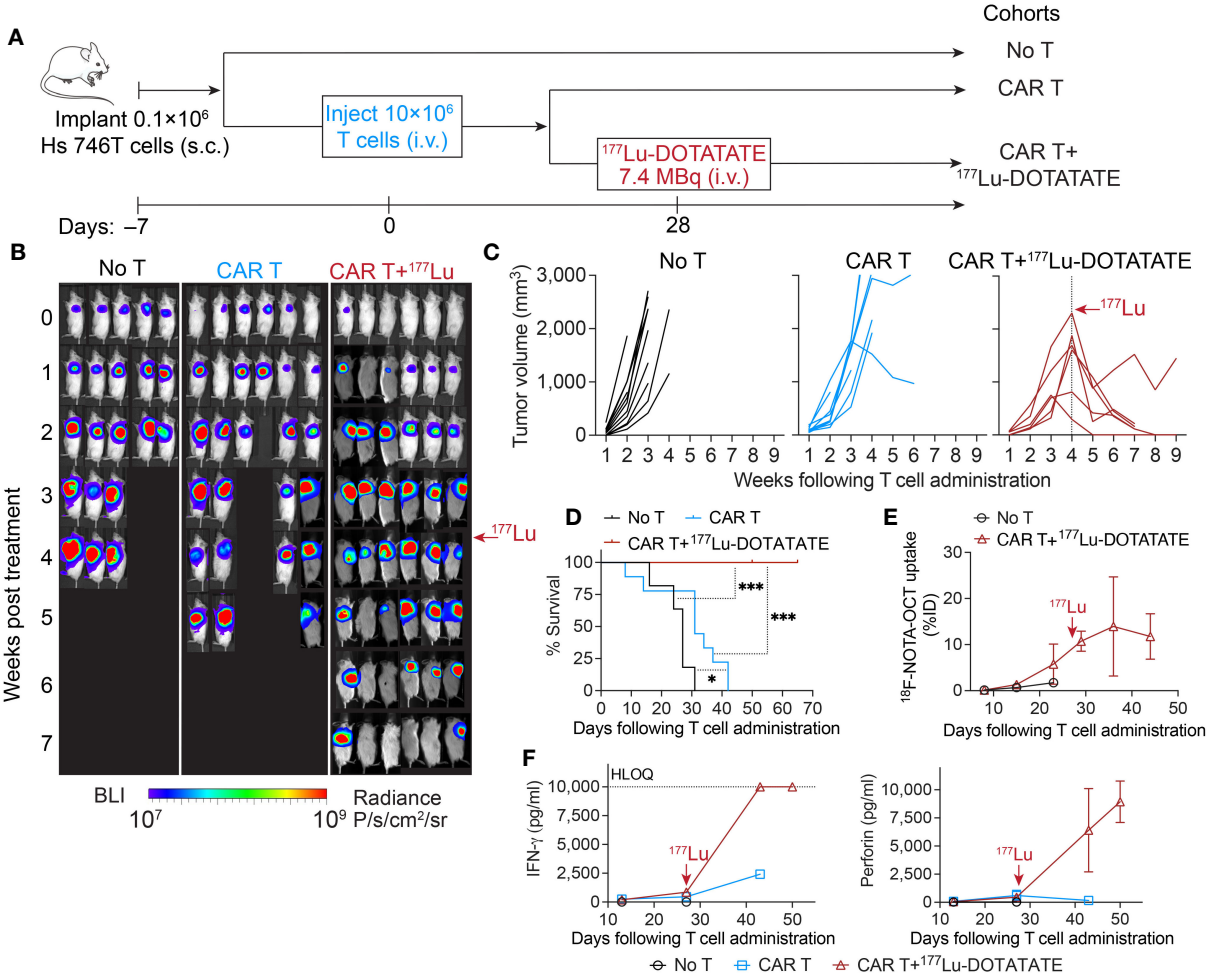
Low-dose ^177^Lu-DOTATATE enhanced antitumor efficacy against established difficult-to-treat gastric tumor. **(A)** Schematic of subcutaneous Hs 746T model. NSG mice bearing subcutaneous Hs 746T tumors were treated with 10 × 10^6^ ICAM-1 CAR T cells 7 days after tumor inoculation. On day 28 post CAR T cell treatment, when significant CAR T accumulation in the tumor was detected by ^18^F-NOTA-Octreotide PET imaging (>3% ID/cm^3^), a subgroup of CAR T-treated mice received a single dose of ^177^Lu- DOTATATE (7.4 MBq). **(B)** Tumor growth monitored by whole-body bioluminescence imaging. **(C)** Tumor volume measurements for individual mice. No T cohort, *n* = 11 biologically independent mice; CAR T cohort, *n* = 8 biologically independent mice (2 T cell donors); CAR T+^177^Lu- DOTATATE cohort, *n* = 6 biologically independent mice (2 T cell donors). **(D)** Kaplan–Meier survival curves. Significance was determined by log-rank (Mantel–Cox) test. *P < 0.05, ***P < 0.001. Data in **C** and **D** are pooled from two independent experiments, each concluded at 7 weeks and 9 weeks post T cell administration, respectively. **(E)** ^18^F-NOTA-Octreotide uptake in tumors determined by PET imaging over time. No T cohort, *n* = 1 biologically independent mouse; CAR T+^177^Lu- DOTATATE cohort, *n* = 3 biologically independent mice. **(F)** Cytokine levels in mouse plasma measured at indicated timepoints post T cell injection. Data represent mean ± SD of 2–3 mice. HLOQ, higher limit of quantification. The red arrows mark the time of ^177^Lu- DOTATATE treatment.

We then set out to investigate the potential of enhancing the anti-tumor immune response through the delivery of low-dose radiation. In the aggressive gastric cancer model of Hs 746T, the activity of ICAM-1 CAR T cells was mostly modest, despite a substantial expansion of CAR T cells within the tumor (**Figures 3A–C**; **1B, C**). Four weeks following CAR T cell injection, a time when significant CAR T expansion was observed in tumor (^18^F-NOTA-Octreotide uptake >3% ID/cm^3^), a subset of mice received a single dose of 7.4 MBq of ^177^Lu-DOTATATE. Impressively, all radiation-treated mice displayed rapid tumor shrinkage and survived significantly longer (**Figures 3B–D**). Within 3–4 weeks of ^177^Lu-DOTATATE treatment, 5 out of 6 mice showed complete tumor regression, while 1 mouse maintained a stable tumor burden after initial tumor reduction (**Figure 3C**). The uptake of ^18^F-NOTA-Octreotide by tumor-infiltrating CAR T cells remained consistently high in the weeks after ^177^Lu-DOTATATE injection (**Figure 3E**), suggesting minimal radiation-mediated CAR T cell death at the dosage of 2.5 Gy. Of note, the elevated levels of IFN-*γ* and perforin detected in mouse serum following ^177^Lu-DOTATATE treatment underscored a radiation-induced effect on T cell immunity (**Figure 3F**).

### 3.4 TRT induces the killing of relapsed gastric tumor

**Figure 4:**
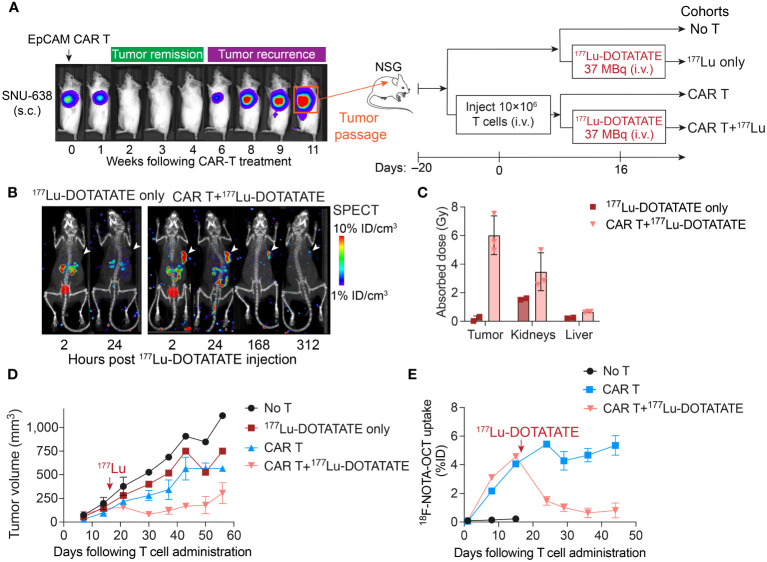
TRT induced the killing of relapsed gastric tumors that were no longer responsive to CAR T cell treatment. **(A)** Schematic of relapsed SNU-638 model. Relapsed SNU-638 tumors were transplanted into NSG mice and treated with 10 × 10^6^ EpCAM CAR T cells 20 days after tumor inoculation when tumors became palpable, or left untreated. On day 16 post CAR T cell treatment, a subgroup of CAR T treated and un-treated mice received a single dose of ^177^Lu-DOTATATE (37 MBq). **(B)** SPECT images demonstrated the selective uptake of ^177^Lu-DOTATATE by tumor-infiltrating CAR T cells, while mice treated solely with ^177^Lu-DOTATATE without CAR T treatment did not exhibit detectable tumor uptake of ^177^Lu-DOTATATE. **(C)** Absorbed doses estimated in tumor, kidneys and liver based on serial SPECT imaging. **(D)** Tumor volume measurements over time. **(E)** ^18^F-NOTA-Octreotide uptake in tumors determined by PET imaging. The red arrows mark the time of ^177^Lu- DOTATATE treatment. Data in **C–E** represent mean ± SD of 2–3 mice.

We have previously developed EpCAM-targeting CAR T cells for the treatment of gastric cancer, which demonstrated potent efficacy in achieving complete responses in subcutaneous gastric cancer models (24). However, tumor recurrence occurred frequently after short duration of complete response. When we transplanted these relapsed tumors into NSG mice and administered fresh EpCAM CAR T cell products, we observed a modest partial response to CAR T cell therapy, despite a substantial expansion of CAR T cells within the tumor. This indicated that the relapsed tumors had acquired resistance, limiting the potential benefits of further CAR T cell treatment. Consequently, we pursued a strategy involving the delivery of higher dose of ^177^Lu-DOTATATE to achieve direct killing of these resistant gastric tumors (**Figure 4A**). The administration of 37 MBq of ^177^Lu-DOTATATE following CAR T cell therapy resulted in absorbed doses of 6.02 ± 1.35 Gy within the tumor, leading to tumor growth control (**Figures 4B–D**). Additionally, SPECT images demonstrated the selective uptake of ^177^Lu-DOTATATE by tumor-infiltrating CAR T cells. In contrast, mice treated solely with ^177^Lu-DOTATATE, without CAR T treatment, did not exhibit detectable tumor uptake of ^177^Lu-DOTATATE (**Figures 4B, C**). Mice that received either CAR T or ^177^Lu-DOTATATE alone experienced tumor progression, although at a slower rate compared to mice receiving no treatment (**Figure 4D**). Longitudinal monitoring of CAR T cells by ^18^F-NOTA-Octreotide PET-CT revealed the simultaneous killing of CAR T cells along with tumor elimination (**Figure 4E**). Similar to the observation in Hs 746T tumor model and ICAM-1 CAR T cells (**Figures 1A–C**), PET-CT imaging showed continuous expansion of CAR T cells as the tumors progressed in mice receiving CAR T cells only (**Figure 4E**). Overall, these findings suggest that TRT holds the potential to effectively eliminate resistant tumors that are unresponsive to CAR T cell treatment.

## 4 Discussion

This study establishes a strategy to deliver internal radiation specifically to tumors by targeting dysfunctional tumor-infiltrating CAR T cells that persistently proliferate upon specific molecular interactions between tumor antigen and CAR molecules. Our findings demonstrate the potential for combining TRT with CAR T cell therapy, which can be tailored to either augment antitumor immunity or achieve maximum killing effects of radiation, while minimizing the risk of systemic toxicity.

The utilization of endoradiotherapy, such as ^177^Lu-DOTATATE in this study, is different from prior efforts on combining external radiation with CAR T cells (7, 8). External radiation is widely accessible and easy to apply, whereas TRT relies on the availability of targetable markers in tumor or tumor stroma. While local external radiation can occasionally induce immune cell-mediated abscopal effects, resulting in the regression of distant disease outside irradiated lesions, the occurrence of such effects is sporadic and unpredictable in patients (32, 33). In contrast, TRT offers the advantage of delivering precise and predictable radiation dosages to multiple tumor lesions, particularly beneficial for patients with metastatic disease. However, very few radiopharmaceuticals have achieved routine clinical adoption because of low therapeutic index. TRT using radiolabeled antibodies is often associated with dose-limiting myelotoxicity and low tumor-to-normal organ ratios (34–36). Low-dose TRT employing ^90^Y-NM600, which targets tumor via lipid rafts, has shown promise in enhancing tumor response to immune checkpoint blockade in preclinical animal models (37). Nevertheless, the notable nonspecific uptake by the liver raises concerns about potential hepatotoxicity.

**Figure 5:**
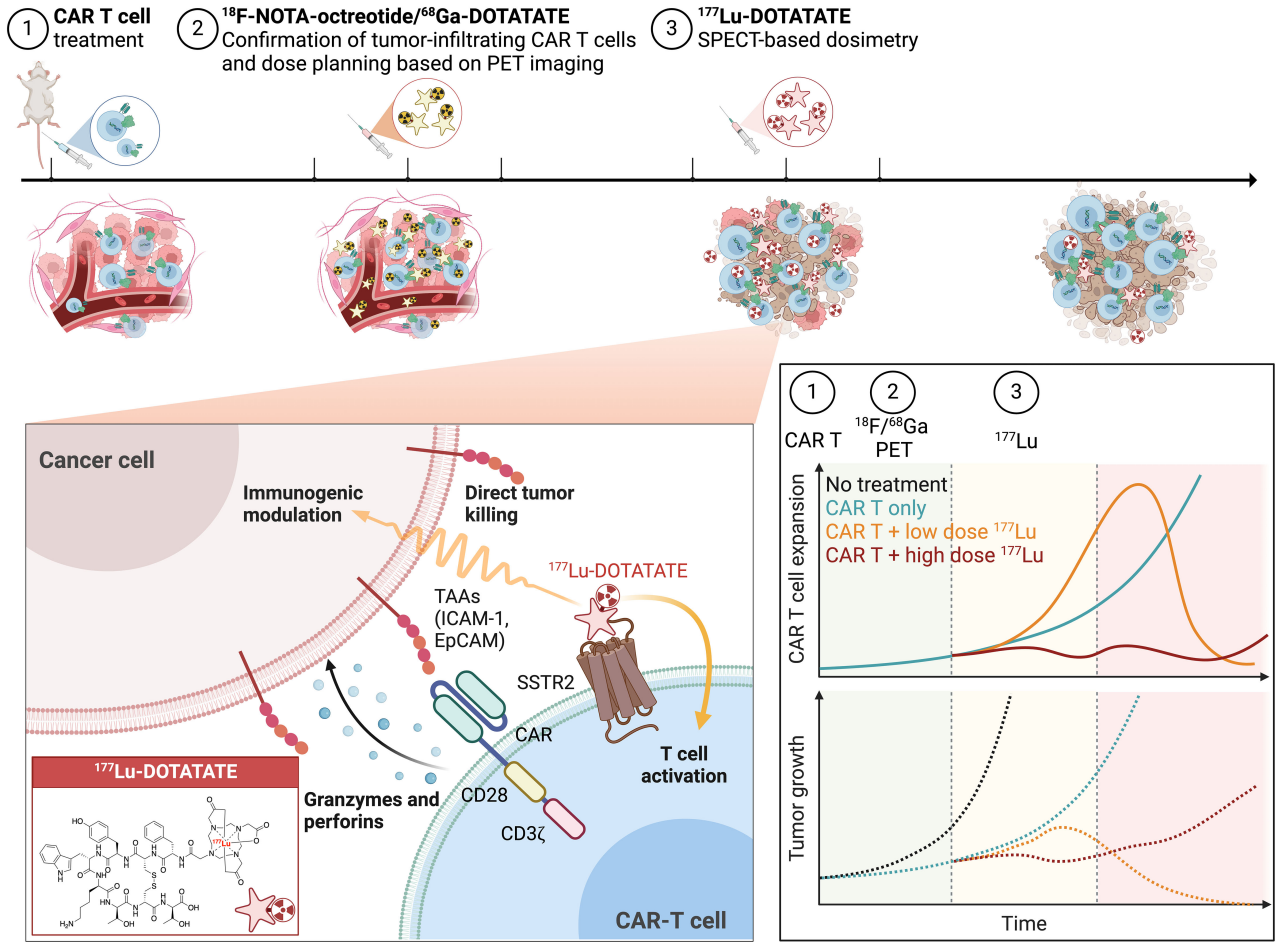
Concept of combinatorial TRT and CAR T cell therapy. The treatment process consists of 3 steps: 1) Infusion of SSTR2-expressing CAR T cells; 2) PET-CT imaging to verify the accumulation of CAR T cells within tumor using either ^18^F-NOTA-Octreotide or ^68^Ga-DOTATATE and employing PET-based dose planning; and 3) ^177^Lu-DOTATATE treatment and SPECT-based dosimetry. The hypothesis is that high-dose TRT can directly induce tumor cell death, whereas low-dose TRT may potentially synergize with CAR T cells to enhance immune responses and produce more enduring tumor control. Figure created with BioRender.com.

Among genetic reporters such as herpes simplex virus type-1 thymidine kinase (HSV1-TK) (38), the human sodium-iodide symporter (hNIS) (39), tPSMA (40), *Escherichia coli* dihydrofolate reductase enzyme (eDHFR) (41), and the anti-benzyl-DOTA scFv (C825) (42), SSTR2 stands out as uniquely well-suited for translational studies of CAR T cell imaging and targeted delivery of therapeutic radionucleotides. This is because of its limited expression in normal organs, which is restricted to neuroendocrine tissues, and the availability of an FDA-approved small-molecule theranostic pair (^68^Ga/^177^Lu-DOTATATE). ^68^Ga/^177^Lu-DOTATATE has demonstrated high specificity to SSTR2, exceptional intratumoral diffusion, favorable renal clearance, and a well-established safety profile. In our phase 1 trial evaluating ICAM-1 CAR T cells against ATC, we successfully utilized ^68^Ga-DOTATATE PET imaging to track CAR T cell kinetics (ClinicalTrials.gov Identifier: NCT04420754), making the first demonstration of noninvasive imaging of CAR T cells in patients (43). The current study investigates the potential of harnessing tumor infiltrating CAR T cells expressing SSTR2 to capture ^177^Lu-DOTATATE for TRT. The treatment process consists of three key steps: 1) infusion of SSTR2-expressing CAR T cells; 2) weekly PET-CT imaging using ^18^F-NOTA-Octreotide to monitor CAR T cell infiltration and expansion within tumors; and 3) ^177^Lu-DOTATATE treatment when sufficient CAR T cell accumulation is observed in tumors (**Figure 5**). Despite the indirect targeting through CAR T cells in contrast to traditional peptide receptor radionuclide therapy (PRRT) directly targeting tumor cells, our system demonstrated remarkable tumor specificity, with minimal activity detected in normal organs at 24 hours post-^177^Lu-DOTATATE injection. The unbound ^177^Lu-DOTATATE molecules, being sufficiently small, undergo rapid clearance from the bloodstream and are excreted via the urinary system. Our system achieved a tumor uptake of 2.4–7.0% ID/g at 24 hours post-injection and a mean tumor absorbed dose of 0.3 Gy/MBq, which are comparable to studies that assessed the direct targeting of neuroendocrine tumor cells overexpressing SSTR2 with ^177^Lu-DOTATATE in animal models (44, 45).

Similar to findings in studies with low–dose external radiation combined with immunotherapy (7, 8, 14), low-dose TRT (~2.5 Gy) delivered via SSTR2-expressing CAR T cells significantly enhanced antitumor activity and induced the expression of the proinflammatory cytokines IFN-*γ* and perforin. In contrast, a moderate dose of TRT (~6 Gy) resulted in killing of resistant solid tumors but simultaneously led to CAR T cell toxicity. These results underscore a clear immunomodulatory effect of low-dose TRT and suggest the presence of an optimum range of TRT dosage for augmenting antitumor immunity. We hypothesize that while high-dose TRT can be employed to directly eliminate tumor cells, low-dose TRT may potentially reprogram the tumor, synergizing with CAR T cells to enhance immune responses and achieve more enduring tumor control (**Figure 5**). It is crucial to conduct further dose-response studies to correlate tumor responses with radiation doses, ranging from immunostimulatory low doses to cytocidal high doses. Additionally, it is important to determine the maximum achievable dose to the tumor, which is associated with the abundance of CAR T cells and targetable SSTR2 molecules within the tumor and is restricted by the maximum acceptable absorbed doses to the kidneys and bone marrow, the dose-limiting organs (46).

Our study presents a proof of concept for a new strategy integrating stimulatory TRT with CAR T cell therapy. Future studies will need to elucidate the underlying mechanism driving this synergistic combination. The major mechanisms of resistance to CAR T cell therapy include tumor antigen loss or heterogeneity, insufficient T cell infiltration, and T cell dysfunction within the tumor microenvironment (TME) (47). Recently, our group conducted single-cell multiomics analysis to assess the crosstalk between CAR T cells and tumors in a gastric cancer tumor model (22). Within resistant tumors, CAR T cells were largely dysfunctional, losing their ability to fight cancer effectively. Specifically, CD8 T cells predominantly showed exhaustion within tumors, while CD4 T cells transitioned into regulatory T cells, dampening the immune response (22). The significant tumor response observed following low-dose TRT might be attributed to enhanced extravasation of non-exhausted CAR T cell into the TME (48) and augmented CAR T cell activity, notably IFN-*γ* production (49), as evidenced in our findings here. Low-dose external radiation has demonstrated to sensitize tumor cells to T cell cytolytic activity through mechanisms involving tumor necrosis factor-related apoptosis-inducing ligand (TRAIL) and Fas ligand (FasL) (7, 8, 50). Moreover, nonablavtive external radiation can induce tumor secretion of chemokines and promote the upregulation of chemokine receptors in tumor-infiltrating CAR T cells and a central memory phenotype (51). However, the biological and immunomodulatory effects of radiation can differ significantly between external radiation generated within minutes and internal radiation persisting for days to weeks. In our forthcoming mechanistic studies, employing a multi-pronged approach that integrates immunohistochemistry, flow cytometry, and single cell multiomics, we anticipate uncovering the phenotypic behaviors and molecular pathways underlying the synergistic interplay between low-dose TRT and CAR T cell therapy. Furthermore, radiotherapy, whether applied externally or internally, has evolved empirically toward radiation doses that maximize tumor cell killing with manageable toxicity to healthy tissues, often without thorough consideration of potential radiation-induced biological effects. Additional studies comparing the biological effects of external radiation versus TRT and their potential for combination with immunotherapies will be valuable in developing optimal treatment strategies.

We recognize the limitations of this study, which include the reliance on human xenograft tumor models that may not fully recapitulate the tumor heterogeneity, tumor stroma, and immune cell interactions that may either augment or dampen CAR T cell activity. Therefore, studies utilizing mouse CAR T cells within an intact immune system and TME using immunocompetent models would provide further support for the potential of this treatment strategy in cancer patients. Secondly, unlike external radiation or TRT targeting tumor directly that can be applied prior to CAR T cell treatment, our approach relies on the homing of CAR T cells in the tumor to capture ^177^Lu-DOTATATE, thus requiring ^177^Lu-DOTATATE injection after CAR T cell treatment. However, it is worth exploring the potential of infusing a second dose of CAR T cells after TRT, which may lead to a superior therapeutic response. Lastly, while our study demonstrated highly specific uptake of ^177^Lu-DOTATATE by tumor-infiltrating CAR T cells and minimal radiation exposure to critical normal organs, thorough assessment of potential toxicities resulting from its combination with CAR T cells is essential. Long-term toxicity concerns also need to be addressed comprehensively.

In summary, we present a translational strategy to augment CAR T cell activity against solid tumors. Further preclinical and clinical studies are necessary to refine the treatment approach and assess the therapeutic efficacy of combining TRT with CAR T cells. In the clinical setting, ^68^Ga-DOTATATE PET-CT or SPECT-CT following an imaging dose of ^177^Lu-DOTATATE can be utilized for dosimetry estimation and to achieve personalized absorbed dose-guided treatment planning. The injection dose of ^177^Lu-DOTATATE can be adjusted to deliver the optimal absorbed dose to the lesion while maintaining acceptable toxicity levels in normal organs. If this combination strategy proves to be beneficial with a tolerable toxicity profile, it may be applicable to CARs targeting other antigens and other types of solid tumors.

## Data availability statement

The original contributions presented in the study are included in the article/supplementary material. Further inquiries can be directed to the corresponding author.

## Ethics statement

The studies involving human specimen were approved by the Weill Cornell Medicine Institutional Review Board. The studies were conducted in accordance with the local legislation and institutional requirements. The participants provided their written informed consent to participate in this study. The animal study was approved by the Institutional Animal Care and Use Committee at Weill Cornell Medicine. The study was conducted in accordance with the local legislation and institutional requirements.

## Author contributions

YY: Conceptualization, Data curation, Formal analysis, Investigation, Methodology, Validation, Visualization, Writing – original draft, Writing – review & editing. YV: Data curation, Formal analysis, Investigation, Methodology, Writing – review & editing. YA: Data curation, Formal analysis, Investigation, Methodology, Visualization, Writing – review & editing. JS: Data curation, Investigation, Visualization, Writing – review & editing. IM: Formal analysis, Methodology, Writing – review & editing. MJ: Conceptualization, Funding acquisition, Investigation, Resources, Supervision, Validation, Writing – original draft, Writing – review & editing.
